# Atoxic Derivative of Botulinum Neurotoxin A as a Prototype Molecular Vehicle for Targeted Delivery to the Neuronal Cytoplasm

**DOI:** 10.1371/journal.pone.0085517

**Published:** 2014-01-22

**Authors:** Edwin J. Vazquez-Cintron, Maksim Vakulenko, Philip A. Band, Larry H. Stanker, Eric A. Johnson, Konstantin Ichtchenko

**Affiliations:** 1 Department of Biochemistry and Molecular Pharmacology, New York University School of Medicine, New York, New York, United States of America; 2 Department of Orthopaedic Surgery, New York University Hospital for Joint Diseases, New York, New York, United States of America; 3 USDA, Agriculture Research Service, Albany, California, United States of America; 4 Department of Bacteriology, University of Wisconsin-Madison, Madison, Wisconsin, United States of America; Institute Pasteur, France

## Abstract

We have previously described genetic constructs and expression systems that enable facile production of recombinant derivatives of botulinum neurotoxins (BoNTs) that retain the structural and trafficking properties of *wt* BoNTs. In this report we describe the properties of one such derivative, BoNT/A *ad*, which was rendered atoxic by introducing two amino acid mutations to the light chain (LC) of *wt* BoNT/A, and which is being developed as a molecular vehicle for delivering drugs to the neuronal cytoplasm. The neuronal binding, internalization, and intracellular trafficking of BoNT/A *ad* in primary hippocampal cultures was evaluated using three complimentary techniques: flow cytometry, immunohistochemistry, and Western blotting. Neuronal binding of BoNT *ad* was significantly increased when neurons were incubated in depolarizing medium. Flow cytometry demonstrated that BoNT/A *ad* internalized into neurons but not glia. After 24 hours, the majority of the neuron-bound BoNT/A *ad* became internalized, as determined by its resistance to pronase E-induced proteolytic degradation of proteins associated with the plasma membrane of intact cells. Significant amounts of the atoxic LC accumulated in a Triton X-100-extractable fraction of the neurons, and persisted as such for at least 11 days with no evidence of degradation. Immunocytochemical analysis demonstrated that the LC of BoNT/A *ad* was translocated to the neuronal cytoplasm after uptake and was specifically targeted to SNARE proteins. The atoxic LC consistently co-localized with synaptic markers SNAP-25 and VAMP-2, but was rarely co-localized with markers for early or late endosomes. These data demonstrate that BoNT/A *ad* mimics the trafficking properties of *wt* BoNT/A, confirming that our platform for designing and expressing BoNT derivatives provides an accessible system for elucidating the molecular details of BoNT trafficking, and can potentially be used to address multiple medical and biodefense needs.

## Introduction

Botulinum neurotoxins (BoNTs) are a family of highly toxic proteins produced by *Clostridium botulinum* [1]–[3]. There are eight BoNT serotypes (A–H) and multiple sub-types, all with common structural features [4]–[7]. Despite their toxicity, BoNTs have become widely used as pharmaceutical agents, because small doses can be applied to paralyze local muscle groups and thereby effect targeted therapeutic paralysis. BoNT/A, with a murine LD_50_ of approximately 0.5 ng per kg, is the serotype most used in clinical medicine (e.g., Ona-, Abo- and Incobotulinum Toxin A, sold under the trade names Botox®, Dysport®, and Xeomin®, respectively) and is approved for a wide range of indications. Other serotypes have also become available for clinical use. The pharmaceutical products primarily differ in purity, stability, and excipient [8]. The unit activity provided in labeling has not been normalized across products, and the approved indications are product-specific. Moreover, because all currently approved pharmaceutical products are wild type (*wt*) toxins derived from the natural *Clostridium botulinum* host, a complex multi-step purification is required, and batch to batch variation with respect to overall content of active protein is difficult to attain.

BoNTs have structural and trafficking features that have ideally evolved for delivery of their metalloprotease entity (light chain, LC) to the neuronal cytosol. They can cross epithelial barriers in the gut and lung, and pass into the circulation. From the circulation, they primarily target active neuromuscular junctions, where they block neurotransmitter release causing peripheral neuromuscular blockade [9], [10]. Death results from respiratory paralysis [2]. All BoNT serotypes have similar structural features, and all target **S**oluble **N**SF **A**ttachment **P**rotein **RE**ceptor (SNARE) components of the molecular machinery for synaptic vesicle release [11]. For example, *wt* BoNT/A is synthesized as a single chain protein, Mr ∼150,000, which is proteolytically activated by an endogenous clostridial protease to generate a heterodimer consisting of a light chain (LC, Mr ∼50,000) and a heavy chain (HC, Mr ∼100,000) linked by an essential disulfide bond [2], [12], [13]. The mature *wt* BoNT/A toxin is a disulfide bonded heterodimer containing three major functional domains: 1) the LC metalloprotease domain responsible for toxicity; 2) the receptor-binding domain comprising the HC C-terminal region (H_C_); and 3) the HC translocation domain comprising the HC N-terminal region (H_N_), which is responsible for the propulsion of the LC to the cytosol [2], [3], [12], [14], [15].

The same multi-step molecular mechanism is responsible for the toxicity and pharmaceutical potency of *wt* BoNT/A, which specifically targets active neurons. This specificity derives from the fact that its receptor, **S**ynaptic **V**esicle protein **2** (SV2), which projects into the lumen of small synaptic vesicles, is only exposed on the plasma membrane during a synaptic vesicle fusion event [16]. The binding and internalization of *wt* BoNT/A also involves gangliosides [12], [17], and immediately after internalization BoNT/A is found in an early endosome compartment [3], [18], [19], which is also associated with synaptic vesicle recycling. Upon acidification of the endosome, BoNT/A undergoes a functionally critical conformational change that enables HC-mediated translocation of the LC into the neuronal cytoplasm [20], [21]. Disruption of the early endosome acidification process by drugs such as bafilomycin or concanamycin A prevents translocation of the light chain to the neuronal cytoplasm [3]. In the neuronal cytosol, the LC, a Zn^2+^-endopeptidase, specifically cleaves **S**y**n**aptosomal-**A**ssociated **P**rotein **25** (SNAP-25), a SNARE protein required for synaptic vesicle exocytosis [15]. Cleavage of SNAP-25 results in inhibition of neurotransmitter release, leading to peripheral neuromuscular paralysis.

Our laboratory has developed a technology platform based on recombinant clostridial constructs, a baculovirus expression system, and purification methods that enable production of recombinant, full-length BoNT heterodimer derivatives [22]. This platform allows the tools of modern molecular biology to be applied to bioengineering of recombinant botulinum neurotoxins that retain the structure and trafficking properties of the native toxin [22], [23]. BoNT/A *ad* (***a***toxic ***d***erivative) is a recombinant derivative of *wt* BoNT/A produced using this platform. This derivative contains functional receptor binding and translocation domains, and an atoxic light chain fused to a sequence representing a cargo site. The BoNT/A *ad* light chain has two mutations introduced into the enzymatic core of the protease, rendering it atoxic. BoNT/A *ad* has an LD_50_ that is 100,000-fold higher than the *wt* toxin. Our previous analysis demonstrated that BoNT/A *ad* accumulates in neuromuscular junctions of the mouse diaphragm after systemic *ip* administration, and can be immunoprecipitated as a complex with SNAP-25 from neuronal cultures [23].

BoNT/A *ad* is designed to enable the LC to safely and efficiently carry cargo to targets associated with the neuronal cytoplasm. In this report, we provide details on the internalization and intracellular trafficking properties of BoNT/A *ad* in primary hippocampal neuron cultures using flow cytometry, immunocytochemistry, and Western blot analysis. We further characterize the neuronal markers associated with the light chain of BoNT/A *ad*, and assess the intracellular stability of internalized LC *ad* in our model system.

## Materials and Methods

### Ethics Statement

Experiments involving animals were conducted with the approval of the New York University School of Medicine (NYU SoM) Institutional Animal Care and Use Committee. NYU SoM animal facilities are maintained in accordance with the Animal Welfare Act, United States Department of Agriculture Regulations (9 CFR, Parts 1, 2, and 3), and the Guide for the Care and Use of Laboratory Animals (National Academy Press, Revised 1996). NYU SoM has a currently approved Animal Welfare Assurance Agreement (No.A3435-01) with the NIH Office for Protection from Research Risks. Veterinary care is provided to animals housed in the animal facilities of the NYU SoM by a veterinarian from the SoM Division of Laboratory Animal Resources. The NYU Medical Center Animal Care & Use Program is fully accredited by the Association for Assessment and Accreditation Of Laboratory Animal Care International (AAALAC).

### Botulinum Neurotoxin A Atoxic Derivative (BoNT/A *ad*)

The full length single chain BoNT/A *ad* was expressed and purified, and then converted to the dichain by treatment with TEV protease as described before [22].

### Preparation and Maintenance of E19 Rat Hippocampal Neurons

Time pregnant Sprague-Dawley rats (Taconic) were used to isolate embryonic-day 19 (E19) hippocampal neurons. E19 rat hippocampal neurons were prepared from hippocampi according to the protocol of Vicario-Abejón [24]. Bilateral hippocampi were dissected from fetal brain, immersed in dissection buffer (15 mM HEPES pH 7.2 (Cat # 15630080, Life Technologies), 0.5% glucose in DPBS without Ca^2+^ and Mg^2+^ (Cat # 14190-250, Life Technologies)), and dissociated by incubation in 10 mL of dissection buffer supplemented with 1x Trypsin/EDTA (10x Trypsin/EDTA is 0.5% trypsin/0.2% EDTA, Cat # 15400054, Life Technologies) for 10 minutes at 37°C. Tissue was triturated using a fire polished Pasteur glass pipette, and cells were counted. The single cell suspension was plated onto poly-L-lysine hydrobromide-coated plates or coverslips in plating medium (1x Minimum Essential Medium-Glutamax™ (1x MEM-Glutamax™, Cat # 41090036, Life Technologies), 10% FBS (Fetal Bovine Serum; Cat # 16000044, Life Technologies), 1x Sodium pyruvate (100 mM Sodium pyruvate; Cat # 11360-070, Life Technologies), 1x Pen/Strep (100x Pen/Strep is 10,000 U/mL penicillin, 10 mg/mL streptomycin; Cat # 15240062, Life Technologies)). After two hours, plating medium was replaced with maintenance medium (1x Neurobasal medium (Cat # 21103049, Life Technologies), 1x B27 supplement (Cat # 17504044, Life Technologies), and 1x Pen/Strep). Three days after plating, 2 µg/mL cytosine β-D-arabinofuranoside (AraC, Cat # C1768, Sigma) was added to the maintenance medium to prevent growth of glia. Half of the medium was replaced with fresh maintenance medium every 3 days. For experiments related to protein quantification by Western blot, 1x10^6^ cells were plated in 100 mm plates in 10 mL medium. For flow cytometry experiments, 3x10^5^ cells were plated in 60 mm plates in 5 mL medium. For immunocytochemical studies, 5x10^4^ cells were plated on cover slips inserted into 6x35 mm/well plates in 3 mL medium/well. Prior to plating, all plates and coverslips were treated with a solution of 10 µg/mL poly-L-lysine hydrobromide (Cat # P2636, MW 30,000–70,000, Sigma) in 100 mM sodium borate, pH 8.5 overnight, washed twice with dissection medium, filled with the appropriate volume of plating medium, and saturated with CO_2_ in a 5% CO_2_ incubator at 37°C for at least 2 hours. The neuronal cultures were used for studies 10 days after plating.

Glass coverslips used for plating neurons for immunocytochemical studies were prepared 2 days before dissection by a 4 hour incubation in *Aqua regia*, consisting of a mixture of 1 part concentrated nitric acid and 3 parts concentrated hydrochloric acid, followed by multiple washes with distilled water. Prepared coverslips were stored in 100% ethanol.

### Western Blot Analysis and Quantification of BoNT/A *ad* LC in Solubilized Samples

BoNT/A *ad* was incubated with neurons for time periods as indicated in figure legends and/or Results. Neurons were harvested and solubilized on ice in 300 µL lysis buffer with protease inhibitors (0.5% Triton X-100, 100 mM NaCl, 25 mM HEPES, pH 7.5, 10 mM 6-aminocaproic acid, 2 mM benzamidine, 5 mM 4-(2-aminoethyl) benzenesulfonyl fluoride hydrochloride (AEBSF), 2.5 mM EDTA, 325 µM bestatin, 35 µM E-64, 2.5 µM leupeptin, 0.75 µM aprotinin) by passing the sample several times through a 25 gauge needle. Soluble protein lyzate was separated from the pellet by centrifuging the samples at 18,000 *g* at 4°C for 20 minutes. For internalization studies, prior to cell lysis, BoNT/A *ad* bound to the cell surface was removed by treatment with 0.1 µg/mL pronase E in DPBS for 5 minutes at room temperature, followed by three washes of the cells with ice-cold DPBS supplemented with 120 mM glucose, 25 mM HEPES, 10 mM 6-aminocaproic acid, 2 mM benzamidine, 1 mM AEBSF, 0.5 mM EDTA, 65 µM bestatin, 7 µM E-64, 0.5 µM leupeptin, and 0.15 µM aprotinin. After lysis, the total protein concentration in each sample was measured and sample volumes were adjusted with lysis buffer, supplemented with protease inhibitors to equalize concentration. Approximately equal amounts (15 µg) of total protein were loaded per lane, separated by reduced SDS PAGE using Criterion 4–12% Bis-Tris precast XT gels (Cat # 345-0124, Bio-Rad), and transferred to a 0.2 µm nitrocellulose membrane (Bio-Rad). Following transfer, membranes were blocked in 10% fat-free milk +5% NGS (Normal Goat Serum, Cat # 10000C, Life Technologies) in TBST (150 mM NaCl, 10 mM Tris-HCl pH 8.0, 0.1% Tween 20) at room temperature for 2 hours. BoNT/A *ad* and *wt* BoNT/A light chains (LC) were probed and visualized with mAb F1-40 (mouse IgG1, final concentration 4 µg/mL). MAb F1-40 is a monoclonal antibody that targets BoNT/A LC provided by Dr. Larry Stanker (USDA). Its properties have been thoroughly described [25]–[27]. Anti-**G**lycer**a**ldehyde 3-**P**hosphate **D**e**h**ydrogenase (GAPDH) mAb (Cat # AM4300, Life Technologies) at a working concentration of 1 µg/mL was additionally used as a normalization control. Anti-mouse HRP conjugated antibodies (Cat # PI-2000, Vector Laboratories, final concentration 35 ng/mL) were used for visualization. Primary and secondary antibodies were diluted in TBST containing 3% NGS. Blots were incubated with primary antibodies overnight at 4°C, and with secondary antibodies 45 minutes at room temperature. Following incubations, blots were washed with TBST 3 times for 5 minutes. Super Signal West Pico chemiluminescent substrate (Cat # 34080, Thermo Scientific) was used for visualization by autoradiography.

Autoradiographs of Western blots were scanned at 300 dpi on an Epson Expression 1680 scanner using Silver Fast AI *v.* 6.4.4r7a software avoiding filter modifications. Band intensities were analyzed with Image J software using a standard algorithm for Western blot analysis. HRP chemiluminescence was measured as a peak area. Samples of BoNT/A *ad* loaded on reduced SDS PAGE with known LC *ad* content (ng/lane) were utilized to generate a standard curve. Samples with Triton-X100 neuronal extracts were normalized to GAPDH loading control, and were quantified as ng LC *ad* per µg total protein.

### Determination of Total Protein Concentration in Solubilized Samples

Total protein concentration in solubilized samples was determined using a Micro BCA kit (Cat # 23235, Thermo Scientific) per the manufacturer’s instructions. Samples were run in triplicate, and the absorbance was measured at 562 nm with a NanoDrop 2000c spectrophotometer. Prior to assay, samples were purified by methanol/chloroform protein precipitation to remove interfering contaminants [28]. Briefly, samples were mixed with 4 sample volumes of methanol followed by 2 sample volumes of chloroform and shaken. Phase separation was accomplished by centrifugation at 18,000 *g* for 5 min. The upper layer was removed, and the interphase protein was precipitated by the addition of 6 sample volumes of methanol followed by 5 min centrifugation at 18,000 *g*. The protein pellet was resuspended and dissolved in 1% SDS in a shaker at 50°C. After determining protein concentrations, samples were normalized using lysis buffer with protease inhibitors.

### Flow Cytometry

BoNT/A *ad* was incubated with neurons for different times as indicated in figure legends or Results. Medium was aspirated; cells were washed three times with room temperature DPBS, harvested, and triturated either with 0.02% EDTA or 1x Trypsin/EDTA (as explained in Results). Cells in suspension were washed with DPBS twice, fixed with 4% formaldehyde (Cat # 15710, Electron Microscopy Sciences) for 15 minutes, permeabilized with 0.1% Triton X-100 (prepared from Surfact-Amps® X-100, Cat # 28314, Thermo Scientific) in DPBS for 5 minutes, washed with DPBS three times, and incubated for 1 hour with anti-LC mAb F1-40 antibody (final concentration 4 µg/mL) diluted in DPBS containing 3% NGS (DPBS-NGS). After incubation, cells were washed twice and incubated for 30 minutes with goat anti-mouse Alexa Fluor® 568 (Cat # A21124, Life Technologies, final concentration 0.8 µg/mL) diluted in DPBS-NGS. After the first round of staining, cells were incubated for an additional 1 hour with mouse anti-GFAP AlexaFluor® 488 mAb (Cat # 53-9892-82, e-Bioscience, final concentration 1 µg/mL) diluted in DPBS-NGS. Cells were washed and resuspended in 350 µl DPBS-NGS. Flow cytometric analysis was performed using an LSR-II customized cell flow cytometer (BD Biosciences). Data were analyzed using *v.*8 FloJo software (Stanford and Tree Star, Inc). The net Median Fluorescent Intensity (MFI) of each sample was calculated as: net MFI = [MFI of cells exposed to BoNT/A *ad*] – [MFI of control cells not exposed to BoNT/A *ad* but treated with the same combination of primary and secondary antibodies].

### Immunofluorescence Analysis

BoNT/A *ad* was incubated with neurons for different times as indicated in figure legends or Results. Immediately after incubation, cells were washed three times with ice-cold DPBS, fixed with 4% formaldehyde for 15 minutes, and permeabilized with 0.1% Triton X-100 for 5 minutes. After fixation the permeabilized cells were washed three times with DPBS, blocked for 1 hour at room temperature with 10% BSA in DPBS, and incubated overnight at 4°C with anti-SNAP-25 (Cat # 111011, Synaptic Systems, final concentration 1 µg/mL), anti-VAMP-2 (Cat # 104211, Synaptic Systems, final concentration 35 ng/mL), anti-EEA1 (Cat # 610457, BD Biosciences, final concentration 0.5 µg/mL), or anti-Rab5 (gift from Dr. Ralph Nixon, NYUSoM, final concentration 4 µg/mL) mAbs. Primary antibodies were diluted in DPBS-NGS. Cells were washed three times with DPBS-NGS and incubated with goat anti-mouse IgG1 Alexa Fluor® 555 secondary antibody (Cat # A21127, Life Technologies, final concentration 0.4 µg/mL) in DPBS-NGS for 45 minutes at room temperature. Since the anti-LC F1-40 mAb is the same isotype (mouse IgG1) as SNAP-25, VAMP-2, and EEA1 mAbs, we directly labeled F1-40 using a Zenon IgG1 Alexa Fluor® 488 labeling kit (Cat # Z25090, Life Technologies) according to the manufacturer’s instructions. After the first round of primary/secondary staining, cells were washed three times with DPBS-NGS and incubated with Zenon-Alexa Fluor® 488 labeled F1-40 for 45 minutes at room temperature. Cells were washed three times with DPBS, and the cover slips were mounted on slides with mounting medium. Image scanning was performed on a Nikon LSM 510 confocal microscope equipped with argon and HeNe lasers producing excitation lines of 488 and 568 nm, and images were analyzed using Zeiss LSM confocal microscopy software (*v.*4.2).

## Results

### BoNT/A *ad* Binding is Neuron-specific and Dependent on Neuronal Activity

**Figure 1:**
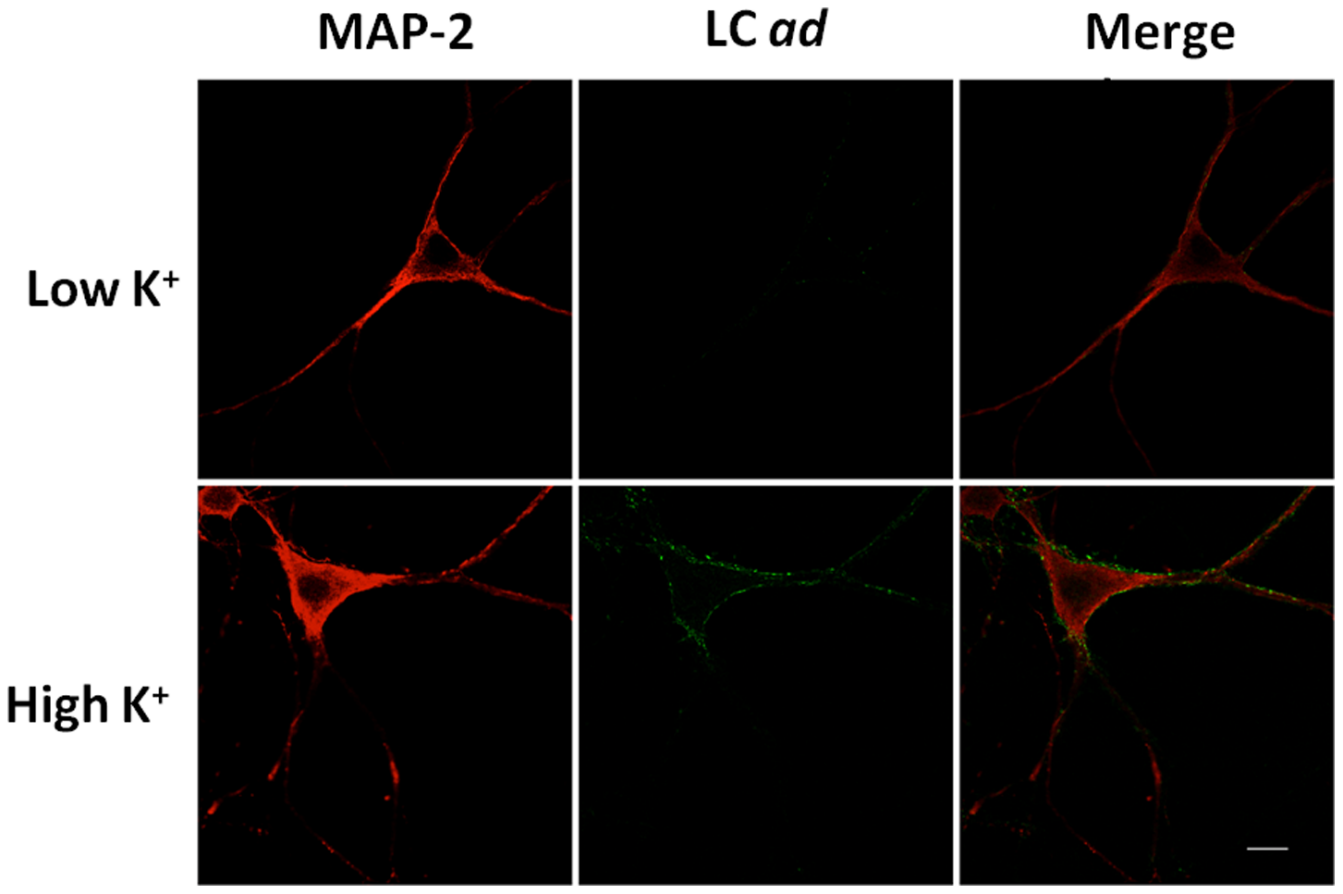
Immunofluorescence analysis showing BoNT/A *ad* binding during neuronal depolarization (active neurons). E19 rat hippocampal neurons cultured in maintenance medium for 10/A *ad* for 1 min at 37°C in HEPES Ringer Resting Buffer (top row) or in high K^+^ HEPES Ringer Depolarization Buffer (bottom row). Anti-MAP-2 chicken monoclonal antibody (Cat # PCK-554P, Covance) was used to stain neuronal soma (red), and F1-40 antibody was used for detection of LC *ad* (green). Scale is 10 µm.

The binding of *wt* BoNT/A to neurons has been demonstrated to occur primarily in neurons that are actively involved in exocytosis because the BoNT/A protein receptor, SV2, is only exposed on the neuronal surface after fusion of synaptic vesicles with the plasma membrane [16]. To confirm that BoNT/A *ad* binding occurs *via* the same mechanism as *wt* BoNT/A, we evaluated the effect of neuronal depolarization on the binding of BoNT/A *ad* to primary rat hippocampal neuron cultures (**Fig. 1**). BoNT/A *ad* binding was visualized with mAb F-140, which recognizes the LC of BoNT/A [25]–[27]. An antibody against MAP-2 was used to visualize the neuronal soma/cytoskeleton and dendrites. The cultures were incubated with BoNT/A *ad* for 1 minute at 37°C in either HEPES Ringer Resting Buffer (HRRB –155 mM NaCl, 5 mM KCl, 2 mM CaCl_2_, 1 mM MgCl_2_, 2 mM NaH_2_PO_4_, 10 mM HEPES, 10 mM (D+)-glucose (Cat # G8769, Sigma), pH 7.2), or HEPES Ringer Depolarization Buffer (HRDB – same composition as HRRB with 56 mM KCl). The high potassium (K^+^) buffer is known to rapidly induce neuronal depolarization. Binding of BoNT/A *ad* was observed in high K^+^ but not in low K^+^ buffer, as previously shown for *wt* BoNT/A [16]. A punctate pattern of BoNT/A *ad* immunofluorescence was observed with F1-40 mAb, consistent with depolarization-induced binding of BoNT/A *ad* to axonal termini making multiple contacts on the surface of the neuronal soma (**Fig. 1**). This demonstrates that BoNT/A *ad* requires neuronal activity for binding.

**Figure 2:**
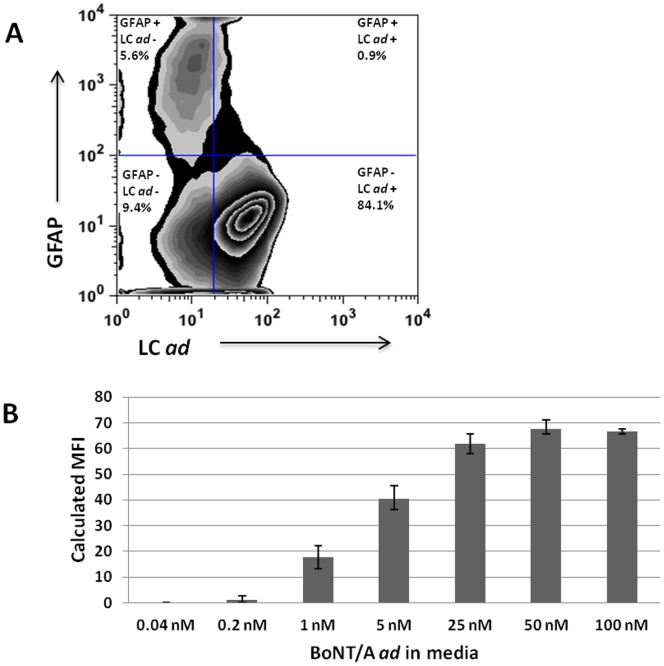
Neuronal uptake of BoNT/A *ad*. E19 rat hippocampal neurons were cultured in maintenance medium for 10/A *ad* for 24 hours at 37°C. Cells were analyzed by flow cytometry. **Panel** **A:** Cells were exposed to 25 nM BoNT/A *ad*. Plot shows cells stained with F1-40 mAb to detect BoNT/A *ad* light chain (X-axis) and with anti-GFAP mAb to detect glia (Y-axis). Numbers in each quadrant represent the percentage of cells in that population. **Panel** **B:** Calculated median fluorescent intensity (MFI) from cell cultures exposed for 24 hr at 37°C to indicated concentrations of BoNT/A *ad*.

Additionally, flow cytometry was used to evaluate the cell specificity of BoNT/A *ad* binding and internalization. In primary hippocampal cultures exposed to 25 nM BoNT *ad* for 24 hrs at 37°C, the majority of cells (84%) associated with BoNT/A *ad* LC (labeled with mAb F1-40) were negative for **G**lial **F**ibrillary **A**cidic **P**rotein (GFAP), a glial marker (**Fig. 2A**). The concentration dependence of BoNT/A *ad* binding and internalization in hippocampal cultures over 24 hours at 37°C is shown in **Fig. 2B**. Binding and internalization were detected in samples incubated with >0.2 nM BoNT/A *ad*, and appeared to reach a plateau at approximately 25 nM. This binding curve was generated from flow cytometric analysis of DAPI-gated neurons as described in **Fig. S1**. BoNT/A *ad* binding and internalization increased between 1 and 24 hours, and to a lesser degree between 24 and 48 hours (**Fig. S1**).

### Cell-associated BoNT/A *ad* Light Chain Accumulates in an Intracellular Compartment

**Figure 3:**
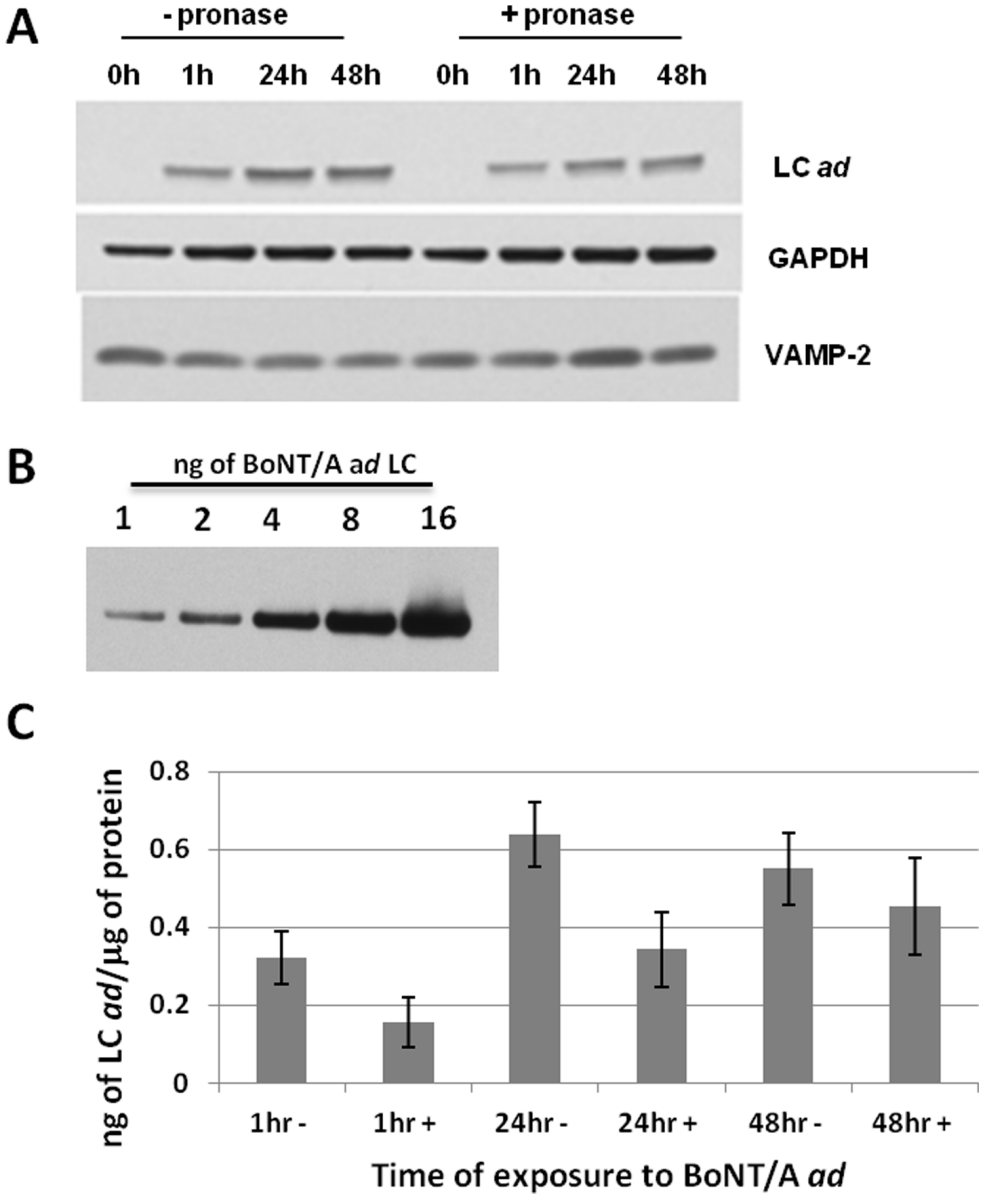
Intracellular accumulation of BoNT/A *ad*. E19.5/A *ad* at 37°C. **Panel** **A:** Western blot analysis after incubation of the cells with BoNT/A *ad* for 1, 24, or 48 hours, followed by treatment with pronase E or control (see Materials and Methods). Protein concentration was normalized to GAPDH. VAMP-2 loading controls were used to demonstrate absence of synaptic protein degradation in experiments with pronase E treatment. **Panel** **B:** Western blot of indicated amounts of BoNT/A *ad* LC developed with F 1-40 mAb. This panel was used to generate the standard curve for LC *ad* quantification. **Panel** **C:** Quantification of the amount of LC *ad* per µg total protein (see Materials and Methods). The experiment was performed in triplicate.

BoNT/A *ad* uptake is a cell-surface receptor-mediated process that involves translocation of the LC to the cytoplasm following receptor binding [12], [14]. Because membrane bound BoNT/A *ad* would be susceptible to extracellular treatment with protease, whereas internalized BoNT/A *ad* would be resistant, we assessed the effect of a brief protease digestion on the amount of BoNT/A *ad* internalized. Neuronal cultures were treated with BoNT/A *ad* for 1 to 48 hours at 37°C. At each indicated time point, the cell cultures were treated with pronase E or vehicle for 5 min at room temperature, similar to the procedure utilized by Francis et al. [29]. The cells were then washed with ice-cold DPBS supplemented with protease inhibitors, solubilized, and extracted with lysis buffer. To determine the percentage of the neuron-bound BoNT/A *ad* that was susceptible to pronase E digestion at each time point, Western blots were developed using mAb F1-40 against LC *ad*. Antibodies against GAPDH were used as a loading control, and antibodies against VAMP-2 were included as an internal control to determine if pronase E treatment affected intracellular SNARE proteins (**Fig. 3A**). A Coomassie-stained gel loaded with an equivalent total protein amount from pronase E-treated and untreated samples is shown in **Fig. S2**, demonstrating similar band pattern from cells treated with BoNT/A *ad* for up to 48 hours, with only a few protein bands disappearing after the five minute incubation with pronase E. To quantitate LC *ad* accumulation, a standard curve of purified reduced BoNT/A *ad* was generated and analyzed by Western blot (**Fig. 3B**). After normalizing each sample to the loading control, the quantity of LC *ad* present in each sample was calculated (**Fig. 3C**). We found that 48% of LC *ad* was resistant to pronase E after 1 hr, 54% after 24 hr, and 83% after 48 hr. This suggests that LC *ad* accumulated in a pronase-resistant intracellular compartment. Intracellular accumulation of LC *ad* increased by approximately 50% from 1 to 24 hr, and by an additional 25% from 24 to 48 hr (**Fig. 3C**). The total amount of BoNT/A *ad* light chain associated with neurons demonstrated apparent saturation after 24 hours of treatment under the conditions tested.

### Calculations of Molarity of LC *ad*, Internalized by Hippocampal Neurons

1. Molecular weight of LC *ad* is 5.2×10^4^
2. Estimated number of harvested neurons (from 1,000,000 plated) is 750,000±200,000 (range: 550,000–950,000)
3. Approximate volume of single pyramidal neuron from rat hippocampus [30], [31] is 4,925±2,505 µm^3^ (range: 2,420–7,430 µm^3^)
4. Approximate volume of all harvested neurons (given by 2×3) is 4.195±2.864 µL (range: 1.331–7.0585 µL)
5. Total protein concentration from Figure 3 is 1279.45±221.05 µg/mL (range: 1,058.4–1,500.5 µg/mL)
6. Total amount of extracted protein in 300 µL of lysis buffer used for harvesting is 426.49±73.69 µg (range: 352.8–500.17 µg)
7. Amount of LC *ad*, associated with total extracted protein is 0.55±0.15 ng/µg (range: 0.4–0.7 ng/µg)
8. Total amount of LC *ad*, associated with total extracted protein (from 6) is 245.62±104.42 ng (range: 141.12–350.12 ng)
9. Concentration of LC *ad* in the total volume of the harvested neurons (from 4) is 77.82±28.21 ng/µL (range: 49.61–106.03 ng/µL)
10. Molarity of internalized LC *ad* (from 1 and 9) is 1.5±0.55 µM (range: 0.95–2.04 µM)

### BoNT/A *ad* Light Chain Co-localizes with SNARE Proteins SNAP-25 and VAMP-2

**Figure 4:**
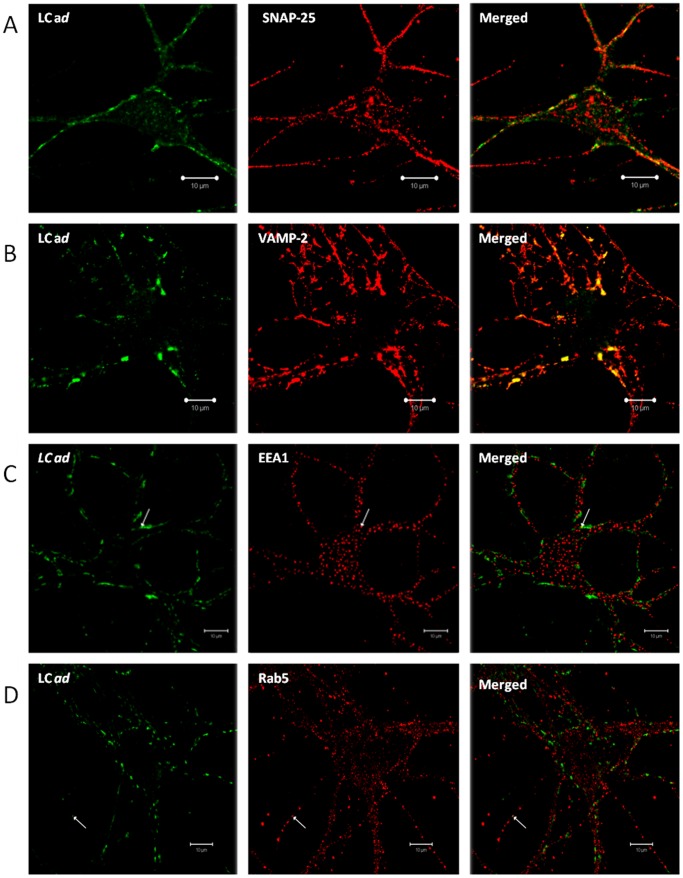
Internalized LC *ad* co-localizes with cytoplasmic/vesicular synaptic markers. E19 rat hippocampal neurons were cultured in maintenance medium for 10°C to 25 nM BoNT/A *ad*. After incubation, cells were washed and processed for immunofluorescence (see Materials and Methods). Cells were stained for LC *ad* and SNAP-25 (**Panel** **A**), VAMP-2 (**Panel** **B**), EEA1 (**Panel** **C**), and Rab5 (**Panel** **D**). Scale is 10 µm.

SNAP-25 and VAMP-2 arehttp://en.wikipedia.org/wiki/N-ethylmaleimide_Sensitive_Factor_or_fusion_protein SNARE proteins that are essential components of the molecular machinery for synaptic vesicle exocytosis, and are exposed to the cytoplasmic compartment of neurons. VAMP-2 is exclusively structurally associated with small synaptic vesicles. SNAP-25 is the molecular target of the LC protease of *wt* BoNT/A [32]. By sequestering and cleaving SNAP-25, *wt* BoNT/A LC prevents synaptic vesicle fusion with the plasma membrane [11]. To determine if internalized LC *ad* is similarly targeted to SNARE proteins, we performed immunohistochemical analysis of BoNT/A *ad*-treated cells. LC *ad* specifically co-localized with SNAP-25 (**Fig. 4A**), the target of the *wt* BoNT/A, and with VAMP-2 (**Fig. 4B**). These experiments confirmed that the LC of BoNT/A *ad* was indeed localized in an intracellular compartment. To determine if some fraction of the LC of BoNT/A *ad* was co-localized with endosomal markers, we performed co-immunostaining with early endosome antigen 1 (EEA1) and the small GTPase Rab5, a marker for an axonal component of early endosome transport. Few EEA1 puncta were co-localized with BoNT/A *ad* LC (**Fig. 4C**). A similar dearth of co-localization was seen with Rab5 (**Fig. 4D**). We also performed co-immunostaining of BoNT/A *ad* LC and the late endosome marker Ras-related protein, Rab7, and similarly, little co-localization was detected (data not shown). In all immunocytochemical staining experiments, the negative controls were neuronal cells that were not treated with BoNT/A *ad*, but which were otherwise identically processed. Negative controls did not stain with anti-LC mAb F1-40, nor with fluorescently labeled secondary antibody (**Fig. S3**). These results indicate that LC *ad* is delivered to the neuronal cytoplasm and targets the machinery for neuronal exocytosis. The data also indicate that very little LC *ad* is sequestered in an endosomal compartment.

### BoNT/A *ad* Light Chain Persistence in Neurons *in vitro*

**Figure 5:**
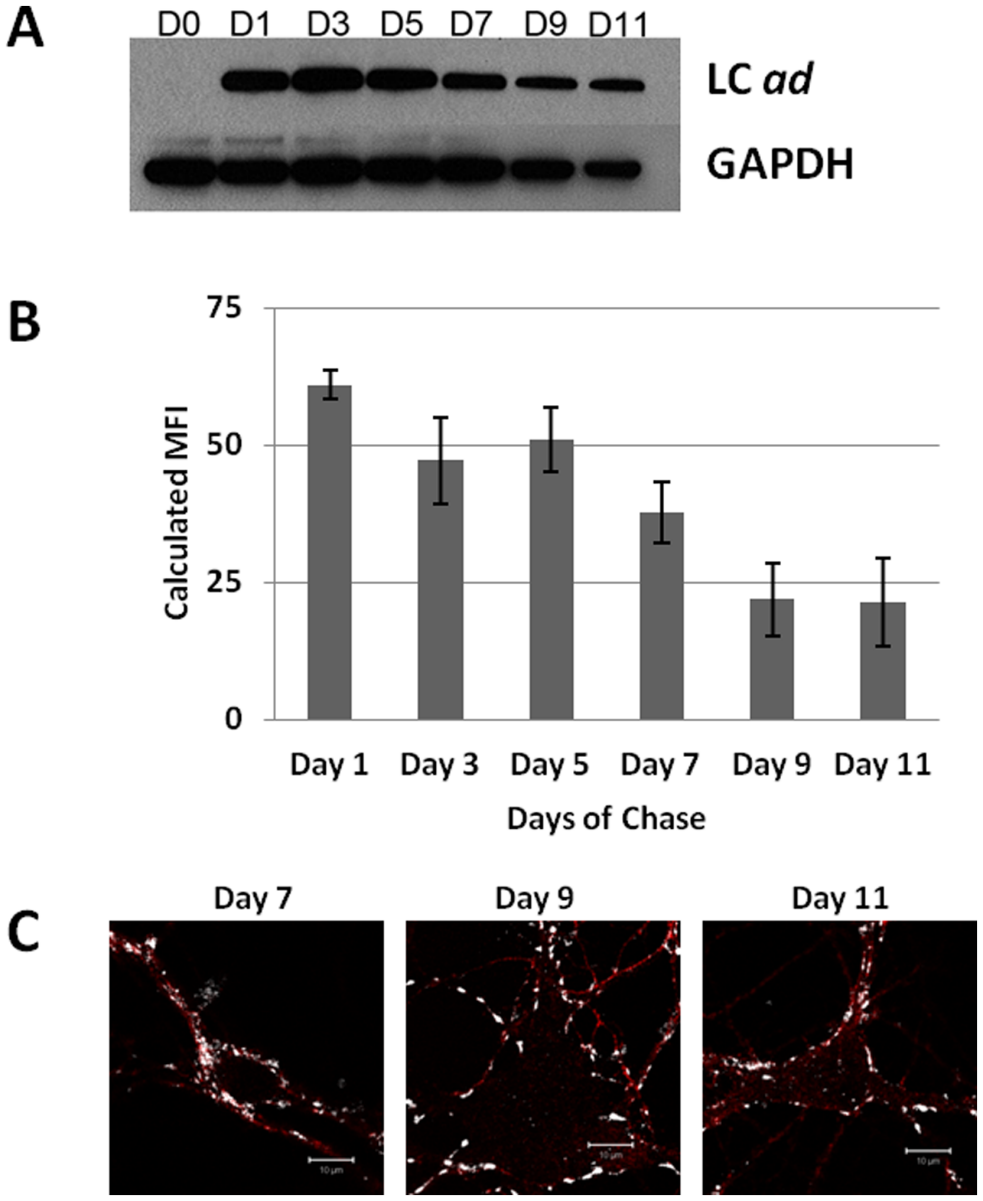
Intraneuronal persistence of LC *ad*. E19 rat hippocampal neurons were cultured in maintenance medium for 10°C to 50 nM BoNT/A *ad*. After incubation, cells were washed twice with maintenance medium to remove residual BoNT/A *ad* and chased with the fresh medium for 1 to 11 days. **Panel** **A:** Western blot analysis of LC *ad* (mAb F1-40). GAPDH was used as a loading control. **Panel** **B:** Flow cytometric quantification of the LC *ad* signal at different days of chase. **Panel** **C:** Immunostaining for *tau* (red, anti-*tau* mouse monoclonal IgG_2b_, Cat # 610672, BD Biosciences) and LC *ad* (white). Scale is 10 µm.

Experiments were performed to determine the persistence of the BoNT/A *ad* LC in the cytoplasmic compartment of hippocampal cultures. Primary hippocampal neurons maintained for 10 days after plating were treated with 50 nM BoNT/A *ad* for 24 hr, after which the cells were washed, and fresh medium was added to the cultures. Cells were harvested 1, 3, 5, 7, 9, or 11 days following media chase and analyzed by Western blot. BoNT/A *ad* LC was detected throughout the 11 days of chase (**Fig. 5A**). No signs of light chain degradation were observed in any of the samples (**Fig. S4**).

To confirm the accuracy of data obtained by Western blot analysis, we prepared identical cultures and analyzed cells by flow cytometry. The relative intensity of the signal from BoNT/A *ad* light chain persisted in a pattern similar to that observed by Western blotting. The amount of LC *ad* recovered from the cells declined after about 7 days of chase, but was relatively stable on days 9 and 11 (**Fig. 5B**). Approximately 50% of the internalized LC *ad* present at day 1 of chase persisted through 8 days. In our hands, and according to others [33], primary hippocampal neurons maintained in Neurobasal medium with B27 serum-free supplement start to exhibit a decline in neuronal survival after three weeks in culture, and therefore, total duration of these experiments was not extended beyond three weeks. Cell viability analysis with propidium iodide showed no signs of primary culture deterioration during the 11-day chase period (data not shown). To further confirm that the intracellular pattern of LC *ad* observed earlier was maintained during the chase, we analyzed cells chased for 7, 9, and 11 days by immunostaining with F1-40 and anti-*tau* mAb. The characteristic punctate pattern of LC *ad* staining was observed at all time points (**Fig. 5C**).

Together, the results of these experiments indicate that LC *ad* accumulates in an intracellular compartment in hippocampal neurons, and persists in that compartment for at least 11 days.

## Discussion

The studies presented here are part of our ongoing effort to produce recombinant derivatives of BoNTs engineered to deliver drugs to the neuronal cytoplasm *via* the trafficking mechanism of native BoNT. All BoNT serotypes specifically deliver their LC protease to the neuronal cytoplasm, and target SNARE proteins. Our approach has been to develop methods to express and purify recombinant derivatives of BoNTs that retain the structure and trafficking properties of the native toxin, but which can be engineered in desirable ways using the tools of modern molecular biology. In this report, we have described the neuronal binding, internalization, and intracellular trafficking of BoNT/A *ad*, a derivative we designed to carry drugs *via* enzymatic coupling to an amino acid residue located at a specific site introduced at the N-terminus of BoNT/A *ad*. The idea is to use BoNT/A *ad* as a “Trojan horse” to deliver therapeutic cargo to intraneuronal targets.

The molecular structure and organization of BoNT/A *ad* provides opportunities to create chimeric fusion proteins to fulfill specific medical needs. BoNT/A *ad* has previously been shown to maintain the structural features and neuronal targeting properties of *wt* BoNT/A both *in vitro* and *in vivo* [22], [23]. Immunocytochemistry experiments presented here demonstrate that BoNT/A *ad* preferentially bound active neurons, and that the LC of BoNT/A *ad* targeted SNARE proteins exposed to the cytoplasm. Flow cytometry experiments demonstrated that BoNT/A *ad* was targeted specifically to neurons but not to glia. Quantitative Western blot experiments demonstrated that the LC of BoNT/A *ad*, the cargo “container”, reached high intra-neuronal levels representing almost 0.05% of the total Triton X-100-extractable protein, and persisted inside neurons for the entire experimental period (11 days). No LC degradation was observed in any of the Western blot experiments. The hippocampal cultures retained their normal appearance and viability throughout the experimental period, suggesting that the large quantity of LC *ad* found inside neurons had no adverse cytotoxic effects.

LC *ad* colocalized extensively with SNAP-25, the substrate of *wt* BoNT/A, and with VAMP-2, the substrate for *wt* BoNT/B, D, F, and G. The punctate LC *ad* pattern observed was consistently localized with synaptic markers exposed to the cytoplasm, rather than being evenly distributed throughout the cell soma. It is particularly noteworthy that the LC of BoNT/A *ad* seldom colocalized with endosomal markers, suggesting that the endosomal compartment is a transient step in BoNT/A *ad* internalization rather than a destination [12]. This contrasts with reports from other laboratories describing delivery vehicles based on engineered derivatives of clostridial toxins, which show endosomal localization and little or no colocalization with synaptic vesicle markers [29], [34]–[38].

The complex structure of BoNTs has made it difficult to produce recombinant derivatives that retain the functional integrity of the *wt* molecule. Therefore, one explanation for the success of BoNT/A *ad* in targeting synaptic markers exposed to the cytoplasm may be the specific expression system and mild two-step purification used for its production. These enable retention of the functional characteristics required for native trafficking. X-ray structural analysis of the *wt* BoNT/A neurotoxin heterodimer shows interaction between multiple amino acid residues of the LC and the belt region of the HC_N_ [12], [20], [39], [40]. Further evidence of the structural importance of LC-HC interactions is the well-known fact that the LC protease is enzymatically inactive as a component of the holotoxin heterodimer, and only becomes active when the LC-HC disulfide bond is reduced. This may also explain the difficulty of reconstituting separated heavy and light chains in a manner that enables LC delivery beyond the endosome [12]. The LC-HC_N_ interaction seen in crystal structures is also essential for the successful translocation of the LC from an acidified endosome to the neuronal cytoplasm [39]. Because the interaction of LC and HC is important to the translocation mechanism itself, preservation of this interaction in BoNT/A *ad,* may explain the successful translocation of the LC *ad* through the endosomal pore.

The amount of the atoxic light chain of BoNT/A *ad* (the “cargo”) that could be delivered to neurons reached a saturable level under the conditions tested. Little additional uptake was seen when BoNT/A *ad* was added to the culture media at concentrations over 25 nM, or when incubation was increased from 24 to 48 hours. We found that approximately 83% of the LC *ad* bound to neurons was internalized by 48 hours. Moreover, the absolute amount of LC *ad* detected intracellularly reached 0.55±0.15 ng per microgram of total Triton X-100-soluble cellular protein, which is equivalent to an intracellular LC *ad* concentration of 1.5±0.55 µM (see Results for detailed calculation). The concentration of intracellular LC *ad* that can be achieved is likely to be useful for drug delivery, especially because no evidence of cytotoxicity was observed in any of our experiments. However, because LC *ad* retains its ability to interact with SNAP-25 [23], this high level of accumulated intracellular LC *ad* may partially block SNAP-25 activity, and thus contribute to the residual toxicity of BoNT/A *ad* (observed at doses 100,000-fold higher than the LD_50_ for *wt* BoNT/A). These properties will have to be considered when developing BoNT/A *ad* to deliver drugs to the cytoplasm of neurons. Work is continuing to identify the cellular components with which BoNT/A *ad* interacts when internalized, and appropriate structural modifications are being tested to further reduce its residual toxicity.

We found that LC *ad* persisted in the neuronal cytoplasm throughout an 11 day chase. These results suggest that the LC *ad* delivered to the neuronal cytoplasm is stable. The persistence of LC *ad* in the neuronal cytoplasm was approximately 100-fold longer than the reported duration of protein delivered to neurons through recombinant tetanus toxin (TeNT) receptor-mediated uptake [29]. We ascribe the stability of LC *ad* in neurons to the fact that LC *ad* was not entrapped in the early endosome after uptake, and was able to undergo endosomal translocation into the cytoplasmic compartment. The short persistence reported for the recombinant TeNT derivative described above [29] might well be explained by its entrapment in the early endosome and subsequent degradation *via* the lysosomal pathway.

We are not aware of any previous attempts to directly quantitate the persistence of *wt* LC by immunohistochemistry or Western blotting, presumably because available methods are not sensitive enough to detect the small quantity of *wt* LC actually persisting in the neurons. The persistence of LC *ad* cannot be directly compared to experiments evaluating the duration of *wt* BoNT/A, estimated to be approximately 11 weeks in culture [19], because the persistence of the *wt* LC has only been evaluated functionally by measuring cleavage of SNAP-25, which can be mediated by a small residual amount of *wt* LC. We can, however, compare our data to experiments performed in neuroblastoma cells treated for 24 hours with 10 nM *wt* BoNT/A, prior to chase. These studies reported a 50% increase in uncleaved SNAP-25 after 8 days of chase, relative to the amount of uncleaved SNAP-25 detected after 1 day [41]. With the rapid turnover of endogenous SNAP-25 (∼16 h in cells actively forming synaptic contacts [42], and assuming a linear relationship between the intracellular concentration of the *wt* LC and the degree of the SNAP-25 cleavage, it would appear that at day 8 (192 h) of chase, the absolute amount of *wt* LC in neuroblastoma cells is likely to have declined by approximately 50%, which is similar to the persistence of the LC *ad* as determined in our experiments.

In summary, the data reported here confirm our previous work demonstrating that our technology platform enables the production of bioengineered recombinant botulinum neurotoxin derivatives that maintain the structure and trafficking properties of *wt* BoNT/A. This platform provides the means to generate BoNTs tailored for specific applications. Here we demonstrate that the atoxic derivative light chain fused with the S6 peptide cargo is delivered to the cytosol of neurons, indicating that this fusion has the potential to be used as a “Trojan horse” to deliver drugs to the neuronal cytosol. BoNT/A *ad* retains the ability to specifically target neurons and to translocate high levels of the LC into the neuronal cytoplasm, where the LC *ad* is able to accumulate and persist without overt evidence of cytotoxicity.

## Supporting Information

Figure S1 BoNT/A *ad* internalization by neurons. Flow cytometry of 10 day old E19 rat hippocampal neurons exposed to 25 nM BoNT/A *ad* for 1, 24, or 48 hours. Cells were stained for BoNT/A *ad* LC with F1-40 mAb and DAPI. **Panel** **A1:** Gating profile of cells based on forward side scatter (FSC) on the X-axis and side scatter (SSC) on the Y-axis. **Panel** **A2:** Gating of cells stained for DAPI. **Panel** **A3:** Cells gated on DAPI^+^ showing presence of BoNT/A *ad* LC: Untreated cells (solid histogram), after 1 hr (dash line), 24 hr (long dash line), and 48 hr (gray dotted line). **Panel** **B:** Analysis of internalized LC *ad* by calculated MFI. Experiments were performed in triplicate.
(TIF)

Figure S2 SDS-PAGE of Triton X-100 extract of hippocampal neurons treated with pronase E and stained with Coomassie brilliant blue. Cells were treated with 25 nM of BoNT/A *ad.* At different time points cells were treated with 0.1 µg/mL pronase E, and proteins were separated by SDS PAGE as described in Materials and Methods. Maintenance media from cells exposed to 25 nM BoNT/A *ad* for 24 hours (+) or not exposed (−) was also subject to SDS PAGE separation.
(TIF)

Figure S3 Negative control of cells stained for BoNT/A *ad*. E19 rat hippocampal neurons were cultured in maintenance medium for 10 days but were not exposed to BoNT/A *ad*. After incubation, cells were washed and processed for immunofluorescence (see Materials and Methods). Cells were stained for LC *ad (green*) MAP2 (red, Cat # PCK-554P, Covance) and *tau* (blue, anti-*tau* mouse monoclonal IgG_2b_, Cat # 610672, BD Biosciences).
(TIF)

Figure S4 Intraneuronal persistence of LC *ad*. Western blot analysis of LC *ad* (mAb F1-40) showing absence of light chain degradation in the samples. E19 rat hippocampal neurons were cultured in maintenance medium for 10 days and then exposed for 24 hours at 37°C to 50 nM BoNT/A *ad*. After incubation, cells were washed twice with maintenance medium to remove residual BoNT/A *ad* and were chased with fresh medium for 1 to 11 days.
(TIF)
