# Supplementary figures and images for: Atoxic Derivative of Botulinum Neurotoxin A as a Prototype Molecular Vehicle for Targeted Delivery to the Neuronal Cytoplasm
Source: PLoS One. 2014 Jan 22;9(1):e85517. doi: 10.1371/journal.pone.0085517 (PMC3899041; doi:10.1371/journal.pone.0085517)

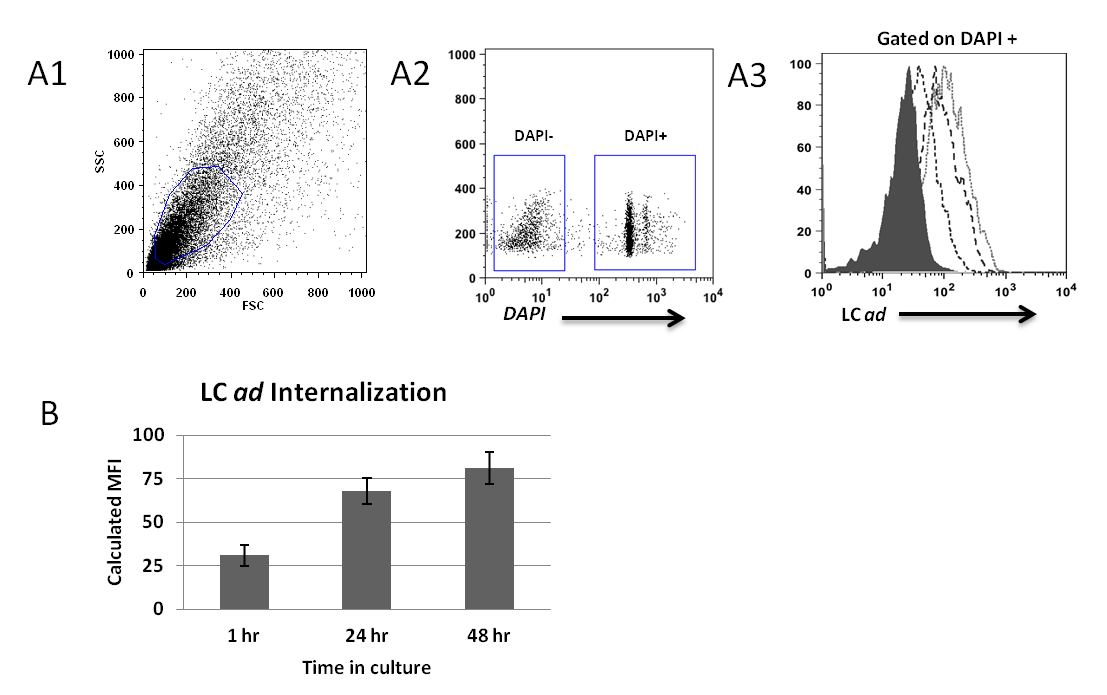

Supplement: Figure S1 — BoNT/A ad internalization by neurons. Flow cytometry of 10 day old E19 rat hippocampal neurons exposed to 25 nM BoNT/A ad for 1, 24, or 48 hours. Cells were stained for BoNT/A ad LC with F1-40 mAb and DAPI. Panel A1: Gating profile of cells based on forward side scatter (FSC) on the X-axis and side scatter (SSC) on the Y-axis. Panel A2: Gating of cells stained for DAPI. Panel A3: Cells gated on DAPI+ showing presence of BoNT/A ad LC: Untreated cells (solid histogram), after 1 hr (dash line), 24 hr (long dash line), and 48 hr (gray dotted line). Panel B: Analysis of internalized LC ad by calculated MFI. Experiments were performed in triplicate. (TIF) [file pone.0085517.s001.tif]

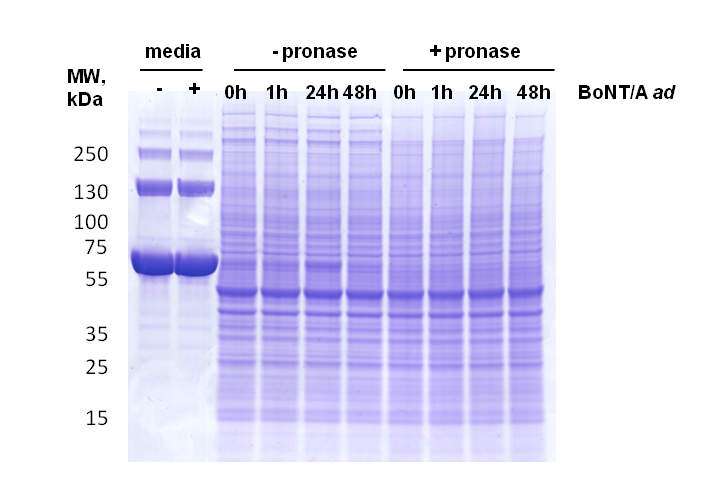

Supplement: Figure S2 — SDS-PAGE of Triton X-100 extract of hippocampal neurons treated with pronase E and stained with Coomassie brilliant blue. Cells were treated with 25 nM of BoNT/A ad. At different time points cells were treated with 0.1 µg/mL pronase E, and proteins were separated by SDS PAGE as described in Materials and Methods. Maintenance media from cells exposed to 25 nM BoNT/A ad for 24 hours (+) or not exposed (−) was also subject to SDS PAGE separation. (TIF) [file pone.0085517.s002.tif]

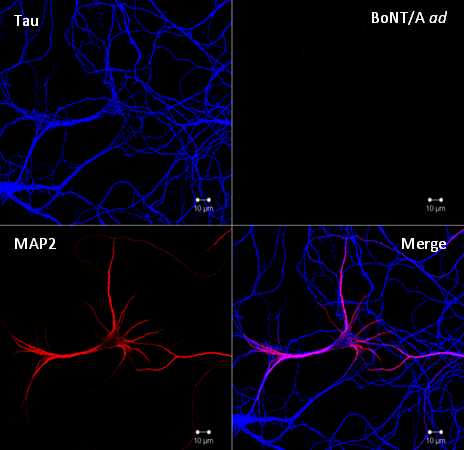

Supplement: Figure S3 — Negative control of cells stained for BoNT/A ad. E19 rat hippocampal neurons were cultured in maintenance medium for 10 days but were not exposed to BoNT/A ad. After incubation, cells were washed and processed for immunofluorescence (see Materials and Methods). Cells were stained for LC ad (green) MAP2 (red, Cat # PCK-554P, Covance) and tau (blue, anti-tau mouse monoclonal IgG2b, Cat # 610672, BD Biosciences). (TIF) [file pone.0085517.s003.tif]

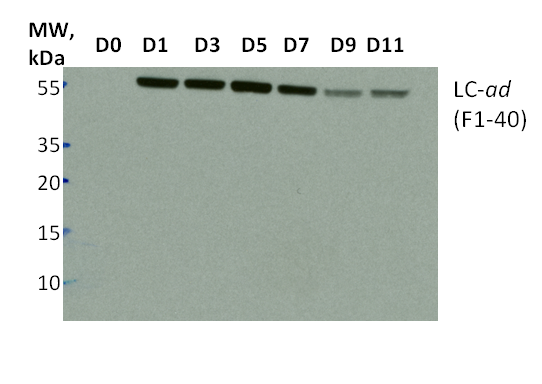

Supplement: Figure S4 — Intraneuronal persistence of LC ad. Western blot analysis of LC ad (mAb F1-40) showing absence of light chain degradation in the samples. E19 rat hippocampal neurons were cultured in maintenance medium for 10 days and then exposed for 24 hours at 37°C to 50 nM BoNT/A ad. After incubation, cells were washed twice with maintenance medium to remove residual BoNT/A ad and were chased with fresh medium for 1 to 11 days. (TIF) [file pone.0085517.s004.tif]
